# Identification of *Philaster apodigitiformis* in aquaculture and functional characterization of its *β-PKA* gene and expression analysis of infected *Poecilia reticulata*

**DOI:** 10.1017/S0031182024000118

**Published:** 2024-04

**Authors:** Chunyu Zhou, Lihui Liu, Mingyue Jiang, Li Wang, Xuming Pan

**Affiliations:** Laboratory of Protozoology, Harbin Normal University, Harbin 150025, China

**Keywords:** infection, *Philaster apodigitiformis*, PKA, *Poecilia reticulate*

## Abstract

Cyclic adenosine monophosphate (cAMP)-dependent protein kinase A (PKA) is a distinctive member of the serine–threonine protein AGC kinase family and an effective kinase for cAMP signal transduction. In recent years, scuticociliate has caused a lot of losses in domestic fishery farming, therefore, we have carried out morphological and molecular biological studies. In this study, diseased guppies (*Poecilia reticulata*) were collected from an ornamental fish market, and scuticociliate *Philaster apodigitiformis* Miao *et al*., 2009 was isolated. In our prior transcriptome sequencing research, we discovered significant expression of the *β-PKA* gene in *P. apodigitiformis* during its infection process, leading us to speculate its involvement in pathogenesis. A complete sequence of the *β-PKA* gene was cloned, and quantified by quantitative reverse transcription-polymerase chain reaction to analyse or to evaluate the functional characteristics of the *β-PKA* gene. Morphological identification and phylogenetic analysis based on small subunit rRNA sequence, infection experiments and haematoxylin–eosin staining method were also carried out, in order to study the pathological characteristics and infection mechanism of scuticociliate. The present results showed that: (1) our results revealed that *β-PKA* is a crucial gene involved in *P. apodigitiformis* infection in guppies, and the findings provide valuable insights for future studies on scuticociliatosis; (2) we characterized a complete gene, *β-PKA*, that is generally expressed in parasitic organisms during infection stage and (3) the present study indicates that PKA plays a critical role in scuticociliate when infection occurs by controlling essential steps such as cell growth, development and regulating the activity of the sensory body structures and the irritability system.

## Graphical abstract

**Figure:**
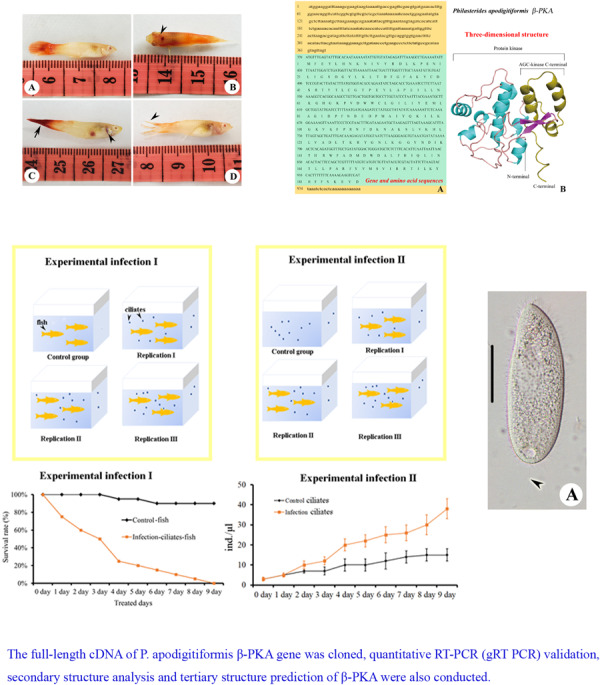

## Introduction

Ciliates which belong to the subclass Scuticociliatia are common in various ecosystems around the world (Thompson and Kaneshiro, 1968; Foissner and Wilbert, 1981; Cawthorn *et al*., 1996; Lynn and Strüder-Kypke, 2005; Fan *et al*., 2011*a*, 2011*b*). They have great species diversity and play different important roles in various ecosystems (Pan *et al*., 2013*a*, 2013*b*; Castro *et al*., 2014; Foissner *et al*., 2014). Many of them are pathogens of invertebrates and fish, which can cause widespread infection of animals in aquaculture, and even cause death in serious cases (Pérez-Uz and Song, 1995; Song and Wilbert, 2002; Fan *et al*., 2009, 2010; Mallo *et al*., 2015).

Scuticociliatosis is caused by parasitic or concurrent parasitic ciliates (Noga, 2010). It is responsible for serious economic losses in commercial fish farms worldwide. In northeastern China, freshwater fish farms are affected by frequent facultative parasitic scuticociliates. Previous studies show that protein kinase A (PKA) is a prominent member of the AGC kinase family. *β-PKA* controls a variety of cellular metabolism processes, including cell growth, gene expression and metabolism (Leroux *et al*., 2018; Kumar *et al*., 2021). Therefore, *β-PKA* can play an important role in various infection and toxicological processes. However, there have been no studies on the role of the *β-PKA* gene in the process of infecting guppy fish by scuticociliate *Philaster apodigitiformis* Miao *et al*., 2009.

In this study, the occurrence of *P. apodigitiformis* outbreaks in *Poecilia reticulata*, alongside a molecular investigation of the *β-PKA* gene, revealed its pivotal role in *P. apodigitiformis* infection within *P. reticulata*. Identification of parasitic *P. apodigitiformis* by traditional morphological methods, haematoxylin–eosin staining and phylogenetic analyses based on the small subunit (SSU) rRNA gene and experimental infection methods were carried out to study its pathological characteristics and infection mechanism. In addition, the *β-PKA* gene of *P. apodigitiformis* was cloned, and its expression was validated by quantitative reverse transcription-polymerase chain reaction (qRT-PCR) after infestation.

## Materials and methods

### Ciliate isolation, cultivation and morphological identification

Diseased fish were selected from an ornamental fish market in Harbin, China, and immersed in sterilized water. Microscopic observation of the water after 1 day revealed the presence of ciliates, which indicated the presence of ciliates in the skin, mucus, gill surfaces and internal organs of the fish. The cells of *P. apodigitiformis* were isolated from infected live fish. A single ciliate was isolated under a dissecting microscope and cultured in sterile RM-9 medium in monoclonal form, maintained at 25°C. Additionally, a ciliate strain of *P. apodigitiformis* (ZCY-20220702) isolated and characterized from the protozoan cell bank of Harbin Normal University was used in the present study. Ciliates were identified by live and post-stained photographs.

### Gene sequencing and phylogenetic analyses

A single cell of *P. apodigitiformis* from the clonal cultures (also established in sterile RM-9 culture medium in a sterile hood and maintained in Petri dishes) was isolated individually using a glass pipette and washed with distilled water. Genomic DNA was extracted from 5 cells using a DNeasy Blood & Tissue Kit (Qiagen, Hilden, Germany), following the manufacturer's instructions. The SSU rRNA gene was amplified with the primers 82F (5′-GAA ACT GCG AAT GGC TC-3′) and 18s-R (5′-TGA TCC TTC TGC AGG TTC ACC TAC-3′) (Medlin *et al*., 1988). The typical amplification profile was programmed as follows: hold at 94°C for 5 min; 35 cycles of denaturation at 94°C for 1 min, annealing at 56°C for 2 min and extension at 72°C for 3 min; and a final hold at 72°C for 10 min. A purified PCR product was inserted into the pUCm-T vector and then sequenced. Bidirectional sequencing was performed at the Shanghai Sunny Biotechnology Company (Shanghai, China). The SSU rRNA gene sequences were compared with other related taxa sequences obtained from the GenBank database on NCBI using the MUSCLE package and were identified as *P. apodigitiformis*, and the newly sequenced SSU rRNA gene of the Harbin population differed from the sequences of previously isolated strains of *Philaster* species by 1–4 nucleotides. Resulting alignments were refined by trimming both ends using BioEdit 7.0.5.2 (Hall, 1999). Bayesian inference (BI) analysis was carried out with MrBayes on XSEDE v3.2.6 (Ronquist and Huelsenbeck, 2003) on CIPRES Science Gateway (Miller *et al*., 2010) using the GTR + I + G evolutionary model as the best-fit model selected by MrModeltest v.2 (Nylander, 2004) according to the Akaike information criterion (AIC), and the support value in BI is called ‘posterior probability’ (PP). A maximum-likelihood (ML) tree was constructed using RAxML-HPC2 v.8.2.10 (Stamatakis *et al*., 2008) on the CIPRES Science Gateway (Miller *et al*., 2010) with the optimal model GTR + I + G evolutionary model as the best model according to the AIC selected by the program Modeltest v.3.4 (Posada and Crandall, 1998). Node support came from 1000 bootstrap replicates. TreeView v.1.6.6 and MEGA v5 were used to visualize tree topologies.

### Histopathology

To confirm the presence of the ciliates, organs and tissues including skin, gills, liver and skeletal muscle of diseased and healthy fish (control group) were isolated and fixed in Bouin's solution for 48 h and maintained in 70% alcohol for gross histopathological analysis. Fixed tissues of fish were sectioned to about 0.6 cm wide slices. Samples were dehydrated in an alcohol gradient, transferred to xylene, embedded in paraffin wax and sectioned at a thickness of 7 μm. Sections were then stained with haematoxylin–eosin and examined under a light microscope.

### Experimental infection

*In vivo* infection experiments were conducted with *P. reticulata*. The average weight of *P. reticulata* individuals was 3 g and the average length was 4 cm. Fish (*n* = 140) were thoroughly examined to confirm that they were free of *P. apodigitiformis* or any other infective agent and cultured in indoor rectangular concrete tanks. The temperature of water was maintained at 25°C, and prior to the scratch, the infection group of fish were anaesthetized with MS-222. Infection by scratch was conducted in tanks containing 2 L of water, 20 fish per tank, in total 1 control and 3 repeats in experiment I, and 3 repeats in experiment II, respectively. For control (experiment II) and each infection groups, about 10 000 (20 mL × 500 ind. mL^−1^) cells of *P. apodigitiformis* were added to each tank. Control treatment in experiment I contained only fish, while control treatment in experiment II contained only *P. apodigitiformis* cells. In the cumulative infection study, the cumulative number of deaths was counted every day. Microscopic examination of wet mounts of skin, gills and internal organs was carried out 7 days post-infection for the cumulative infection study. The animal experiments were conducted following the Guide for the Care and Use of Laboratory Animals, and the protocol for which was approved by the Harbin Normal University.

### RNA isolation, PCR amplification of the coding region of the *PKA* gene

*Philaster apodigitiformis* was cultured to a concentration of about 100 ind. mL^−1^. After enriched several times to obtain 100 μL of ciliate solution, RNA was extracted using a RNA Extraction Kit (TransGen Biotech, Beijing, China) according to the manufacturer's instructions, and the resulting RNA was reverse transcribed to cDNA using the Reverse Transcription Kit M-MLV reverse transcriptase (TaKaRa, China) according to the manufacturer's instructions. The *β-PKA* gene was amplified using PCR. PCR primers are listed in Table 1. PCR conditions were as follows: 3 min at 94°C, 2 cycles of 15 s at 95°C, 2 min at 52°C and 2 min at 72°C, 2 cycles of 15 s at 95°C, 2 min at 54°C and 2 min at 72°C, 35 cycles of 15 s at 95°C, 2 min at 58°C and 2 min at 72°C and 72°C for 10 min.

**Table 1.** Primer sequence and their applications

Primers	Sequences (5′–3′)	Applications	*T*_m_ (°C)
PKA-792F	TTCATTCGGTCGTGTTCG	cDNA cloning of gene	52.5
PKA-792R	GGCTTGTAAGTTGGTGGG	cDNA cloning of gene	53.7
PKA-5A-491	GGCTTGTAAGTTGGTGGG	5′ RACE (1st round PCR)	53.7
PKA-5B-226	TTAGGATACCAAGGCACCAC	5′ RACE (2nd round PCR)	54.2
PKA-5C-196	GCTTGCCGTGACCTTTA	5′ RACE (3rd round PCR)	52.6
PKA-3-206-A	GGTGGTGCCTTGGTATCCTAA	3′ RACE (1st round PCR)	56.9
PKA-3-443-B	TGGACTGGGATGCTCTCTTTCACA	3′ RACE (2nd round PCR)	60.2
18S-qpcr-F	CCTGGGAAGGTACGGGTAAT	qPCR (*β*-actin mRNA)	56.5
18S-qpcr-R	AAGGTTCACCAGACCATTCG	qPCR (*β*-actin mRNA)	54.9
PKA-F-138	TATGGACTGGGATGCTCTCT	qPCR (PKA mRNA)	54.8
PKA-R-138	TCATCAGCAGGTTTAATTGGTTTAG	qPCR (PKA mRNA)	52.6

### cDNA synthesis and 5′, 3′ RACE

The extracted RNA was reverse transcribed using the 3′-Full RACE Core Set with PrimeScriptRTase (TaKaRa, China) according to the manufacturer's instructions. The *β*-*PKA* gene was amplified using PCR. PCR primers are listed in Table 1. PCR contained reactions I and II. In PCR I, the DNA double helix is denatured, usually accomplished by heating the sample to a high temperature (around 94–98°C), which results in the separation of the double-stranded DNA into 2 single strands. In PCR II, the DNA polymerase extends the primers by adding nucleotides to produce a new complementary strand of DNA, and this process results in the synthesis of 2 new strands of DNA, each complementary to one of the original DNA strands. PCR I was performed as follows: 94°C for 5 min, 40 cycles (94°C for 30 s, 55.7°C for 30 s and 72°C for 60 s), and 72°C for 5 min in a 25 μL PCR volume. PCR II was performed as follows: 94°C for 5 min, 40 cycles (94°C for 30 s, 59.1°C for 30 s and 72°C for 60 s), and 72°C for 5 min in a 25 μL PCR volume.

### Gene expression analysis by qRT-PCR

qRT-PCR was performed as described in previous studies. Briefly, after RNA isolation and reverse transcription, an UltraSYBR mixture (Beijing ComWin Biotechnology Co., Ltd, Beijing, China) was used, with a CFX96 multicolour real-time PCR detection system (Bio-Rad Laboratories, USA). The temperature was first maintained at 95°C for 30 s, followed by 40 PCR cycles at 95°C (15 s) and 60°C (1 min) to obtain a melting curve from 60 to 95°C. qRT-PCR data were analysed using *β*-actin as an internal reference gene, as described in our previous study. The primers used to detect the expression of the *β-PKA* and 18S rRNA (internal reference gene) genes are shown in Table 1. The 2^−ΔΔCT^ method was used to calculate the relative expression of each gene.

### Secondary structure analysis of *β-PKA* gene

The structure of PKA protein was predicted using Alphafold v2.2.0, and the results after plotting graphs with PyMOL follows the analysis of Wang *et al*. (2022).

## Results

### Pathological features and infectivity

#### Pathogenicity/histopathology

**Figure 1.**
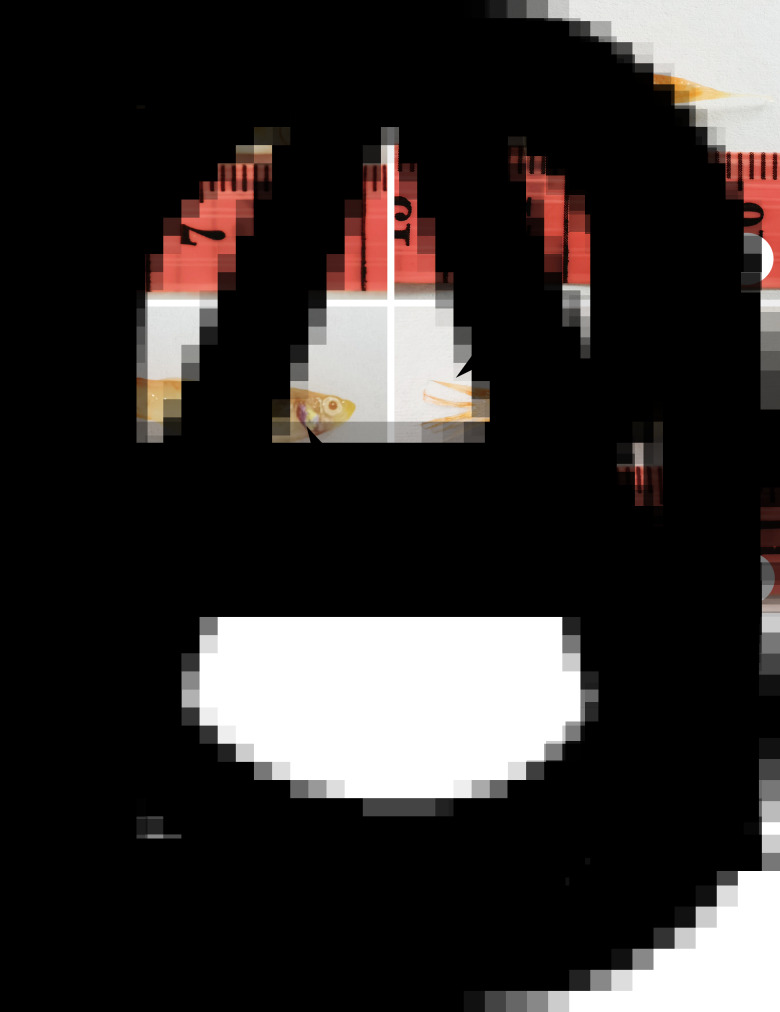
Pathological alterations in *Poecilia reticulata*: (A) a healthy *P. reticulata* individual; (B) a conspicuous bleeding spot directly above the fish's head; (C) highlights evident of bleeding spots within the abdominal cavity and (D) a visibly fractured and forked tail fin.

**Figure 2.**
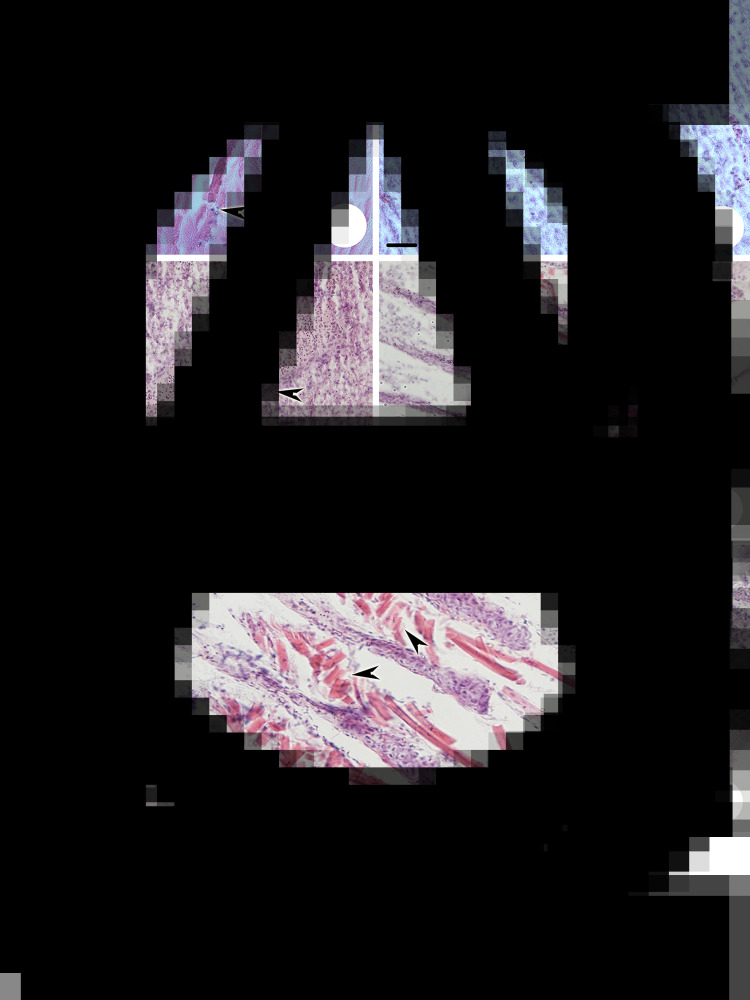
Histological sections of *P. reticulata* stained with haematoxylin–eosin: (A) deformed and disordered arrangement of muscle tissue (arrowheads), (B) disordered arrangement of liver tissue (arrowheads), (C) shrunken and deformed spleen tissue (arrowheads) and (D, E) shrunken and cracked gill filaments (arrowheads). Scale bar = 200 μm.

On first inspection the guppies did not have any apparent gross signs of scuticociliate infection. Nonetheless, the parasite was observed in fresh-mount preparations on the skin, in the gills and in internal organs. The diseased fish showed varying degrees of lesions including skin, eyes, caudal fins, spleen and liver. There were bleeding spots and black or grey lesions randomly distributed on the body surface of diseased fish (Fig. 1B and C; Table 2). The black or grey lesions were necrotic areas containing *P. apodigitiformis* Miao *et al*., 2009. Lesions caused by *P. apodigitiformis* destroying the fish fins and the regular fish were intact (Fig. 1A) but broken to varying degrees in diseased fish (Fig. 1C and D). Gills of all diseased fish were shrivelled, dark and anaemic, and several had dark-red ulcerations. Some cells of *P. apodigitiformis* were detected in gaps among the gill filaments. All the fish were examined histologically by haematoxylin–eosin staining. As shown in Fig. 2, the skeletal muscle, liver, gill and spleen of the fish were damaged to varying degrees. Scrapings of these lesions revealed the presence of *P. apodigitiformis.*

**Table 2.** Characterization of different groups of guppies infected by *P. apodigitiformis* (unit: ind.)

Groups	Loss of scales	Skin necrosis (grey)	Skin necrosis (white)	Fin bifurcation	Haemorrhagic (fish body)	Haemorrhagic (fish head)
Experiment I	18	14	16	18	12	11
Experiment II	20	22	28	29	11	12

#### Description of morphology of *P. apodigitiformis*

**Figure 3.**
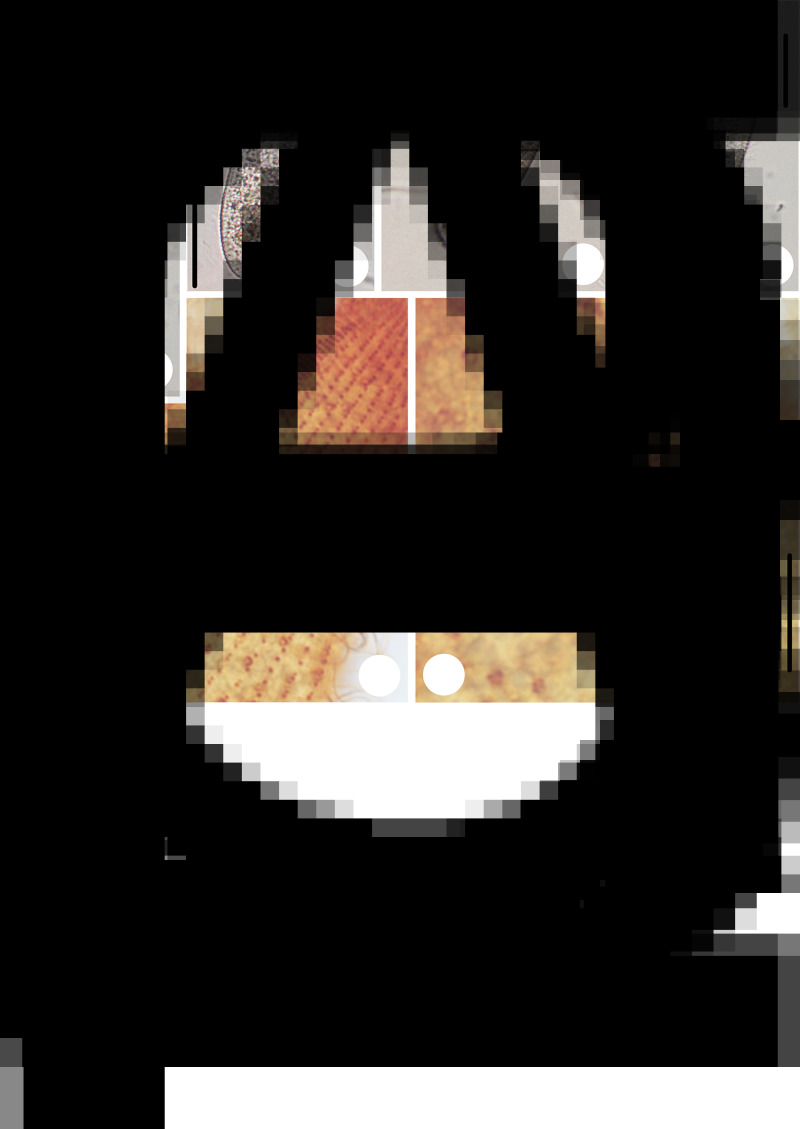
Morphology and infraciliature of *Philaster apodigitiformis* Miao *et al*., 2009: (A–D) ventral views of different individuals *in vivo* and (E, F) infraciliature. Scale bars: 15 μm.

Size *in vivo* about 40–60 × 15–25 μm^2^ (Fig. 3A–D; Table 3). Body shape elongate and tapering slightly towards anterior (Fig. 3A–D; Table 3). No apical plate. Buccal field extending to about 30% of body length, with shallow depression. Pellicle smooth, with bar-shaped, very fine, short (2 μm long) extrusomes. Cytoplasm colourless to greyish, containing several food vacuoles and bar- or dumbbell-like crystals (Fig. 3B–D; Table 3). Single macronucleus centrally located, spherical to ovoid. One contractile vacuole positioned caudally. Somatic cilia densely arranged, approximately 5 μm long; 1 caudal cilium about 10 μm in length (Fig. 3A, arrowhead; Table 3). Movement with no special features, swimming moderately fast. Somatic kinetics composed of dikinetids, approximately 35 (Fig. 3E and F; Table 3). Membranelle 1 triangular, consisting of 7 rows of basal bodies; membranelle 2 containing about 20 rows; membranelle 3 short and containing 3 rows of basal bodies (Fig. 3E and F; Table 3). Paroral membrane terminating at anterior edge of membranelle 3. Scutica, with about 5–7 basal bodies, arranged in long line (Fig. 3E and F; Table 3). The SSU rRNA gene sequences of Harbin population of *P. apodigitiformis* has been deposited in the GenBank database with the accession number OR642804.

**Table 3.** Morphometric characterization of *Philaster apodigitiformis*

Character	Min	Max	Mean	*M*	s.d.	CV	*n*
Body length	43	68	52.2	51	6.7	12.8	21
Body width	18	27	23.4	22	3.7	15.8	21
Number of somatic kinetics	31	37	35	35	1.4	4	15
Length of buccal field	15	18	16.2	16	0.7	4.3	12
Macronucleus, length	7	11	8.1	8	0.8	9.9	19
Macronucleus, width	6	10	7.9	7	0.4	5.1	18

CV, coefficient of variation (%); *M*, median; Max, maximum; Mean, arithmetic mean; Min, minimum; *n*, number of specimens; s.d., standard deviation.

Data from stained specimens.

### Phylogenetic positions of *P. apodigitiformis*

**Figure 4.**
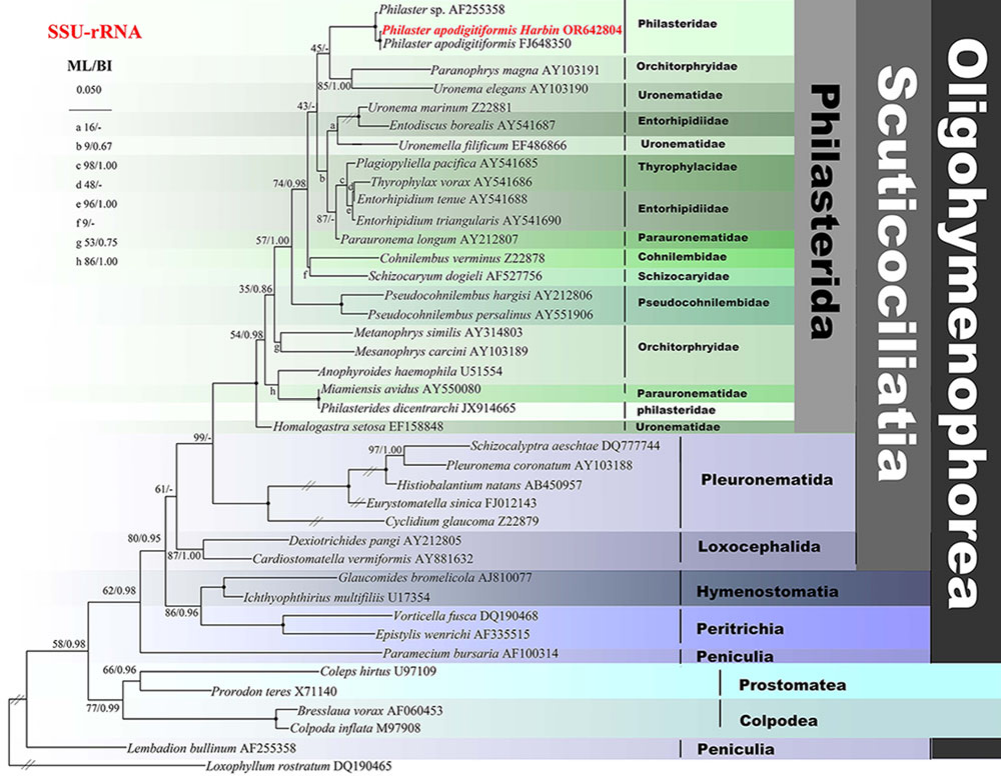
ML tree inferred from the SSU rRNA gene sequences, showing the positions of *P. apodigitiformis* (in bold). Numbers at nodes represent the bootstrap values of ML out of 1000 replicates and the PP of BI. Fully supported (100%/1.00) branches are marked with solid circles. The scale bar corresponds to 5 substitutions per 100 nucleotide positions.

The ML and BI trees have almost identical topologies, therefore only the ML tree is shown (Fig. 4). Figure 4 shows that the Harbin population of *P. apodigitiformis* groups with *P. apodigitiformis* FJ648350 with full support and the clade of which then groups with *Philaster* sp. with full support.

#### Experimental infection

Following Crosbie *et al*. (2012) and Ravindran *et al*. (2022), we used Koch's postulates to evaluate the hazards of ciliated infected fish, that is: (1) the same parasite can be discovered in every infected fish but not in the healthy individual, (2) isolate such parasite from the host and establish a pure culture in the medium, (3) the same disease will repeat itself by inoculating a healthy and sensitive host with a pure culture of this parasite and (4) the parasite can be isolated and cultured from the host, where the same disease is tested.

**Figure 5.**
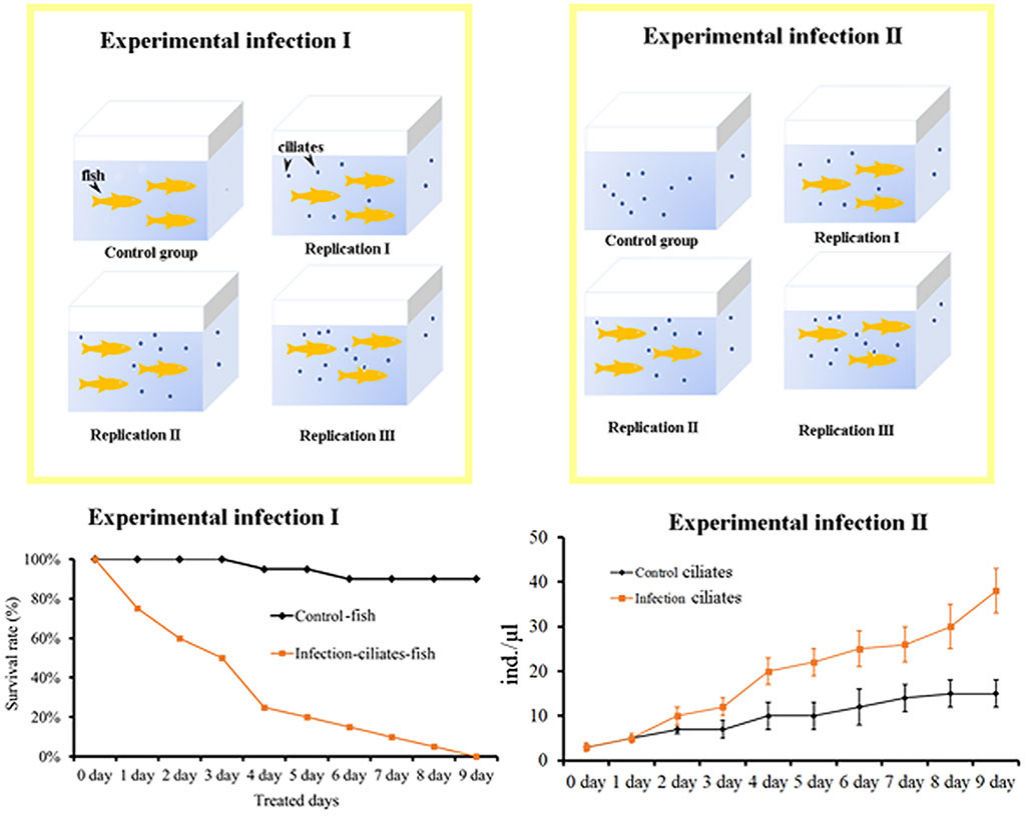
Details of the experimental set-up.

We designed experiment I to verify that *P. apodigitiformis* was the parasite that causes disease and experiment II to investigate expression of *β-PKA* of *P. apodigitiformis* during infection (Fig. 5). In the infection experiments I and II, fish infected with *P. apodigitiformis* stayed upside down with mouth opening, hardly breathing, secreting a lot of mucus and exhibiting blackness of the body. Six repeat groups of scratched fish infected with *P. apodigitiformis* randomly showed black or grey skin lesions, and the infected fish had uniformly different degrees of ulceration, in addition to the obvious increase in mucus on the surface of the fish. These symptoms are very similar to those of the natural forms of the disease. The fish in the experimental group began to die 1 by 1 since the first day, and until the 9th day, all individuals (*n* = 20) had died. The number of *P. apodigitiformis* in the experimental group was significantly increased compared to the control group in all cases.

### Sequence analysis of *PKA* gene

**Figure 6.**
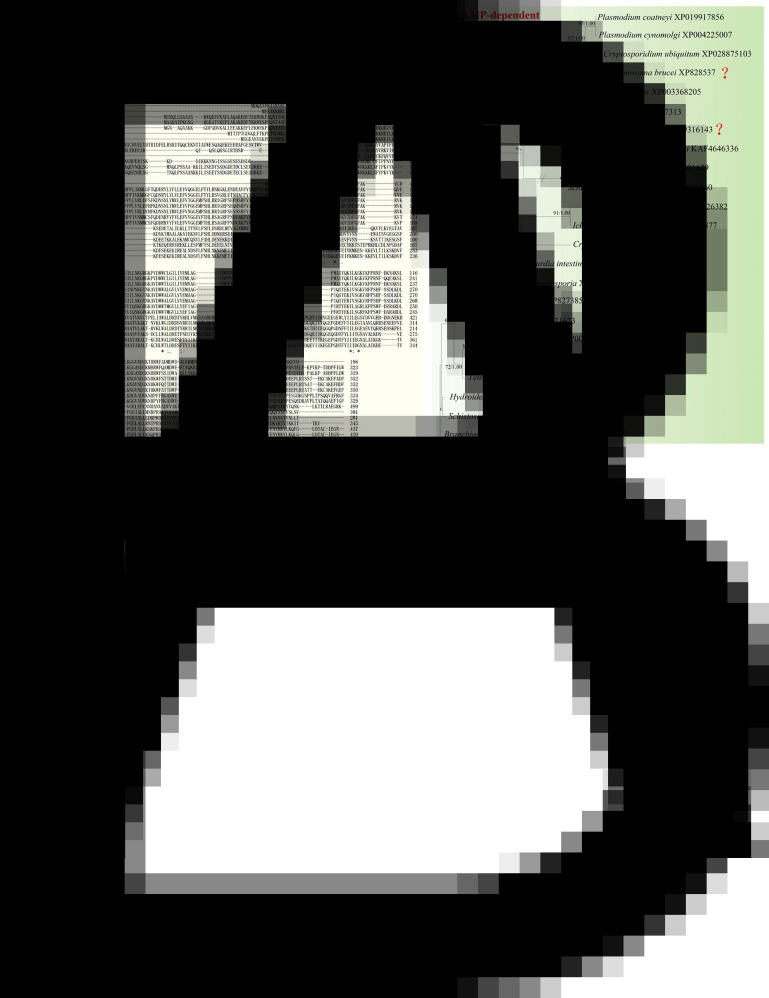
(A) Full-length gene and amino acid sequences of PKA of *P. apodigitiformis* and (B) predicted 3-dimensional conformational map of *PKA* gene protein. The lowercase letters in the figure represent the UTR region, the uppercase letters are the coding region gene sequences and the separate uppercase letter represents the translated amino acid.

The full-length cDNA of PKA is 957 bp, including an open reading frame (ORF) of 588 bp, which encodes a polypeptide (PKA) consisting of 196 amino acids with a predicted molecular weight of 22.97 kDa (Fig. 6A). In total, 369 bp were obtained by 5′-RACE amplification, whereas 24 bp by 3′-RACE amplification. Using BLAST analysis and comparison of sequences available in the NCBI database, it was confirmed that the novel nucleotide sequence belonged to *β-PKA*. The full-length cDNA sequence of *β-PKA* spanned 957 bp, of which the ORF spanned 588 bp, the 5′-untranslated region (UTR) 369 bp and the 3′-UTR 24 bp. In addition, the poly (A) tail of the 3′-UTR sequence composed of 12 nitrogenous bases.

### Secondary structure analysis of *β-PKA* gene

As shown in Fig. 6B, it is found that the secondary structure of PKA protein is mainly *α*-helix, containing a small amount of *β*-fold structure (20–23, 26–30), and the secondary structure is consistent with the usual characteristics of kinases. In terms of primary sequence, according to the annotation of homologous proteins in the UniProt protein structure database (https://www.uniprot.org/uniprotkb/I7MM26/entry#family_and_domains), the PKA proteins belong to the protein kinase structural domain, and the C-terminal amino acids 145–196 belong to the AGC-kinase C-terminal structural domain, which is essential for the development of kinase function.

### Phylogenetic analysis of PKA protein

**Figure 7.**
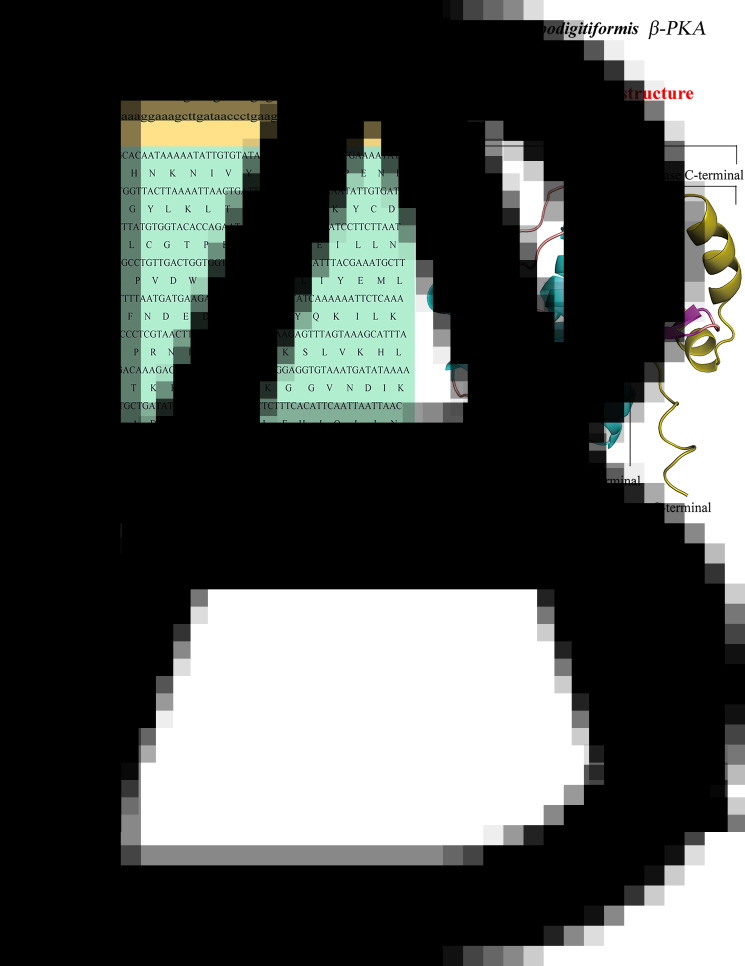
(A) Multiple alignments of amino acid sequences of *PKA* gene from different species and (B) ML tree inferred from *PKA* gene protein showing the systematic position of *P. apodigitiformis* (in bold). Bootstrap values above 50 for the ML (1000 replicates) and/or BI (1000 replicates) are given at the individual nodes. Scale bar corresponds to 10 substitutions per 100 nucleotide positions.

ML and BI trees showed nearly identical topologies, therefore only the ML tree is shown in Fig. 7. *Branchiostoma floridae* was used as outgroup. We have acquired nearly all available sequences of the PKA protein to present a comprehensive overview of PKA protein for parasites. Several parasitic ciliates (*P. apodigitiformis*, *Tetrahymena thermophila* and *Paramecium tetraurelia*) group together with strong support, the group of which then clusters with other parasites, e.g. *Hydroides elegans*, *Plasmodium falciparum*, *Schistosoma haematobium*, *Trypanosoma brucei brucei* and *Trypanosoma cruzi* with high support values (Fig. 7). Notably, parasitic ciliates divide into 2 groups, 1 contains *P. apodigitiformis*, *T. thermophila* and *P. tetraurelia*; the other includes *T. thermophila* and *Ichthyophthirius multifiliis*. In specific branches, particular sequences (indicated by question marks) fail to cluster with closely related ones. This occurrence might stem from misidentification or the potential that they are paralogous sequences. Consequently, we opted to remove these questionable sequences during the alignment process, thereby ensuring that the number of taxa in the alignment matches that of the tree.

### *PKA* gene expression in *P. apodigitiformis*

**Figure 8.**
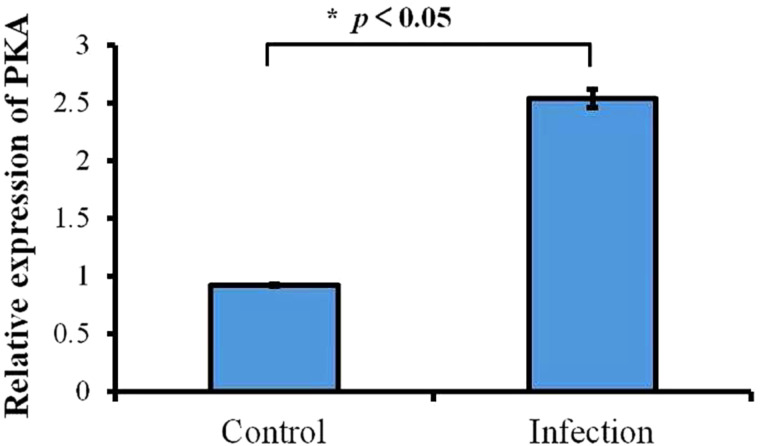
Relative expression profile of *PKA* gene mRNA was determined by qRT-PCR for non-infection and infection groups. Data relative to mRNA levels were normalized by *β*-actin and presented as the mean ± s.e.m.

For control and each infection groups, about 10 000 cells were added. During infection, the PKA mRNA level in the *P. apodigitiformis* increased significantly, compared with the non-infection (control) group (*P* < 0.05, Fig. 8).

## Discussion

### Comments on morphology and systematic position of *P. apodigitiformis*

The most important criteria for species identification and separation in the genus *Philaster* are oral membranelles 1–3, ratio of the length between body and oral field and the number of somatic kinetics (Miao *et al*., 2009). Harbin population of *P. apodigitiformis* only differs from the population described by having a smaller body size (40–60 μm × 15–25 μm *vs* 60–90 μm × 30–40 μm). Harbin population of *P. apodigitiformis* groups with *P. apodigitiformis* FJ648350 with full support, which supports the morphological identification of this species (Fig. 4).

### Pathological features of *P. apodigitiformis*

Histopathological studies of scuticociliatosis have been carried out and reported in detail many times, especially in fish (Jin *et al*., 2000; Iglesias *et al*., 2001; Harikrishnan *et al*., 2010, 2012). The most obvious feature of the diseased fish is that the lesion area becomes white, slightly swollen and soft to touch, and this feature is more obvious in young fish. In adult fish, the whitish area eventually becomes congested and ulcerated. In addition to the skin, fins and muscles on the surface of the fish's body, scuticociliate can also invade the abdominal cavity, heart, brain and other internal organs and tissues (Kim *et al*., 2006). In contrast to tetrahymenosis, scuticociliatosis occurs mostly in marine aquaculture (e.g. *Stichopus japonicus*, *Scophthalmus maximus* and *Brachyura*) and does not have a wider range of hosts (e.g. slugs, chick embryos, dragonflies, helgramites, roaches, cockroaches and caterpillars) (Li *et al*., 2021). In addition to this, scuticociliates mainly affects marine fish and *P. apodigitiformis* affects freshwater fish (Lom and Dyková, 1992; Thilakaratne *et al*., 2003; Imai *et al*., 2009; Harikrishnan *et al*., 2010). In our study, we confirmed that scuticociliate could also affect freshwater fish, e.g. guppy.

The results of the survival rate test indicated that *P. apodigitiformis* was the direct cause of death of guppy, and *Philaster* species is an opportunistic invader that is potentially harmful to aquaculture. *Philaster* species can undergo mass reproduction under suitable conditions. In the initial stage of the infection test, the density of the ciliates was 5 ind. mL^−1^ (10 000 cells added into 2 L water) in each group. In the later stage, the densities of the *Philaster* species in the infection groups reached ca. 5000 ind. mL^−1^. To reduce these issues in fish cultures, it would be beneficial to change a significant amount of water during the initial stage of infection, remove dead fish and minimize any mechanical damage to the fish.

### Analysis of *PKA* gene and PKA protein in *P. apodigitiformis*

The *PKA* gene has been reported in previous studies to have the ability to participate as a second messenger and to help *Plasmodium* invade the host cell (Flueck *et al*., 2019; Patel *et al*., 2019; Kumar *et al*., 2021). In the previous study, we found a significant upregulation of the expression level of *β-PKA* gene by the transcriptome analysis after the pre-infestation of *P. apodigitiformis* on ornamental fish. In the present study, we cloned the complete sequence of *PKA* gene in *P. apodigitiformis*, and measured its expression by qRT-PCR.

The PKA pathway is one of the most versatile and best studied signalling pathways in eukaryotic cells (Søberg *et al*., 2017). Previous studies confirmed that protein kinases exerted major regulatory effects in eukaryotic signalling events, and PKA was a regulatory protein that plays a crucial role in signal transduction and signalling pathways (Eisenhardt *et al*., 2001; Fischer *et al*., 2003). The present study indicates that the cyclic adenosine monophosphate-dependent protein kinase plays a critical role in controlling essential steps such as ciliates’ growth, development and regulating the activity of the sensory body structures and the irritability system of parasitic scuticociliates.

Thus, in order to verify the role of the *β-PKA* gene in *P. apodigitiformis* during infection, we cloned and expressed the complete sequence of *PKA* gene. A 957 bp sequence was obtained, and the ORF encoded 196 amino acid residues. Moreover, the identified amino acid sequence encoded by the *PKA* gene of *P. apodigitiformis* was highly similar to those of other species, e.g. *H. elegans*, *P. falciparum*, *S. haematobium*, *T. brucei brucei* and *T. cruzi* (Fig. 7B). In addition, analysis of the predicted tertiary structure of the protein revealed that PKA protein mainly contained *α*-helix and a small amount of *β*-fold structure (20–23, 26–30). The secondary structure is consistent with the usual characteristics of kinases, which indicates that the *β-PKA* gene is relatively stable, conserved and showed high homologous. Meanwhile, our results show that the expression level of *PKA* gene is significantly increased during infection. The present study suggests that PKA can be used to explore in greater depth the capabilities exerted during infection.

## Data Availability

The data that support the findings of this study are available from the corresponding author upon reasonable request.
